# Neuropharmacological effects of *Nigella sativa*

**Published:** 2016

**Authors:** Farimah Beheshti, Majid Khazaei, Mahmoud Hosseini

**Affiliations:** 1 *Departments of Physiology, School of Medicine, Mashhad University of Medical Sciences, Mashhad, Iran*; 2 *Neurogenic Inflammation Research Center, School of Medicine, Mashhad University of Medical Sciences, Mashhad, Iran*; 3 *Neurocognitive Research Center, School of Medicine, Mashhad University of Medical Sciences, Mashhad, Iran*

**Keywords:** *Nigella sativa*, *Thymoquinone*, *Central nervous system*, *Neuropharmacological effects*

## Abstract

*Nigella sativa* (NS) (Ranunculaceae family) is generally utilized as a therapeutic plant all over the world. The seeds of the plant have a long history of use in different frameworks of medicines and food. In Islamic literature, it is considered as one of the greatest forms of therapeutics. It has been widely used to treat nervous system diseases such as memory impairment, epilepsy, neurotoxicity, pain, etc. Additionally, this is uncovered that the majority of therapeutic properties of this plant are due to the presence of thymoquinone (TQ) which is a major bioactive component of the essential oil. Pharmacological studies have been done to evaluate the effects of NS on the central nervous system (CNS). The present review is an effort to provide a detailed scientific literature survey about pharmacological activities of the plant on nervous system. Our literature review showed that NS and its components can be considered as promising agents in the treatment of nervous system disorders.

## Introduction

Medicinal plants have been utilized in the treatment of ailments for many years in different aboriginal medicine as well as folk medicine. Furthermore, therapeutic plants are additionally utilized as a part of the arrangement of home-grown pharmaceuticals as they are thought to be safe as to modern medical cares (Darakhshan et al., 2015 ▶; Gilani et al., 2001 ▶). Among different therapeutic plants, *Nigella sativa*(NS), from Ranunculaceae family, normally develops in Eastern Europe, Middle East, and Western Asia. NS seed is known as ''Al-Habba Al-Sauda'' and Al-Habba Al-Barakah'' in Arabic and black seed or dark cumin in English (Gilani et al., 2004 ▶). For many years, NS seeds have been reported to be utilized as a remedy for various ailments in the Middle East and some Asian nations (Bailey & Day, 1989 ▶). This plant is particularly important in Islamic nations, due to its numerous useful properties (Khare, 2004; Al-Ghamdi, 2001 ▶). Moreover, NS seed can lessen weakness and depression and improve the body's vitality in the Avicenna's well known book ''Canon of Medicine'' (Yarnell & Abascal, 2011 ▶). This plant has been included in the list of natural medications in different medicines including Tibb-e-Nabavi (The medication of Prophet Mohammad), Unani Tebb and Indian system of medicine (Rajsekhar & Kuldeep, 2011 ▶; Razavi & Hosseinzadeh 2014 ▶).

NS has been widely investigated for its biological activities and restorative potential and demonstrated to have a wide range of activities such as diuretic, anti-hypertensive, anti-diabetic, anti-cancer, immune-modulatory, antimicrobial, anthelmintic, analgesics and calming, spasmolytic, bronchodilator, anti-inflammatory, anti-tussive, gastro-protective, hepato-protective, low density lipoprotein cholesterol decreasing, renal-protective and anti-oxidant properties (Abel-Salam, 2012 ▶; Keyhanmanesh et al., 2014a ▶;Keyhanmanesh et al., 2014b ▶; Kapoor, 2009 ▶; Hanafy & Hatem 1991 ▶; Abdel-Daim MM &Ghazy EW, 2015 ▶; Mousavi & Mohajeri, 2014 ▶; Pourbakhsh et al., 2014 ▶; Gholamnezhad et al., 2014 ▶; Dahri et al., 2005 ▶). In traditional medicine, the seeds of NS are generally utilized as a part of the treatment of different illnesses like obesity, back pain, hypertension and gastrointestinal problems, bronchitis, asthma, cardiac diseases, sexual diseases, diarrhea, rheumatoid arthritis and skin disorders (Goreja, 2003 ▶; Boskabady et al., 2011a ▶; Boskabady et al., 2011b ▶; Al Mofleh et al., 2008 ▶; Ali et al., 2008 ▶). It is likewise utilized as a liver tonic, digestive, food craving stimulant, emmenagogue, carminative and diuretic agent and in order to enhance milk production in nursing moms, and for treatment of delayed menses (Abel-Salam, 2012 ▶; Atta, 2003 ▶). The greatest part of the remedial properties of this plant is due to the presence of thymoquinone (TQ) which is a major active chemical component of the essential oil (Darakhshan et al., 2015 ▶). Black seeds are also used in food as flavoring additive in the bread and pickles, because it has very low level of toxicity(Al-Ali et al., 2008 ▶; Randhawa & Alghamdi, 2011 ▶).

**Characteristics of** ***Nigella*** **sativa**

NS is a yearly blooming plant which grows up 20-90 cm tall, with finely isolated leaves and the leaf fragments are barely straight to threadlike (Sharma et al., 2009 ▶). The flowers are delicate and generally hued white, yellow, pink, light blue or pale purple, with 5-10 petals (Ismail, 2009 ▶). The natural fruit is an expansive and expanded capsule made out of 3-7 united follicles, each containing numerous seeds (Al-Ali et al., 2008 ▶).

Visibly, seeds are small dicotyledonous, trigonus, angular, regulose-tubercular, 2-3.5mm×1-2mm, dark externally and white inside, smell marginally fragrant and taste bitter (Paarakh, 2010 ▶). Microscopically, transverse segment of seed shows single layered epidermis comprising of oval, thick walled cells, covered externally by a papillose cuticle and filled with dark brown contents (Chatap et al., 2006 ▶). Epidermis is trailed by 2-4 layers of thick-walled tangentially extended parenchymatous cells, trailed by a rosy chestnut pigmented layer made out of thick-walled, rectangular amplified cells (Türkdoğan et al., 2001). Inward to the pigment layer, there is a layer that is made out of thick-walled rectangular stretched or almost columnar, extended cells (Elmansy & Almasry, 2013). Endosperm comprises of thin-walled, rectangular or polygonal cells mostly loaded with oil globules (Paarakh, 2010). The microscopy of seed powder shows earthy dark, parencymatous cells and oil globules (Warrier et al., 2004).

Numerous active compounds have been derived, distinguished and reported so far in distinctive mixtures of dark seeds. The most imperative active compounds are TQ (30%-48%), thymohydroquinone, dithymoquinone, p-cymene (7%-15%), carvacrol (6%-12%), 4-terpineol (2%-7%), t-anethol (1%-4%), sesquiterpenelongifolene (1%-8%), α-pinene and thymol and so forth (Shrivastava, Agrawal, & Parveen, 2011). The seeds also contain two separate sorts of alkaloids i.e. isoquinoline alkaloids e.g. nigellicimine and nigellicimine N-oxide, and pyrazol alkaloids or indazole ring bearing alkaloids which incorporate nigellidine and nigellicine (Khan et al., 2003). NS seeds additionally contain alpha-hederin, a water soluble pentacyclic triterpene and saponin, a potential anticancer agent (Al-Jassir, 1992 ▶; Ansari et al., 1988 ▶).

The seeds of NS also contain protein (26.7%), fat (28.5%), carbohydrates (24.9%), crude fiber (8.4%) and total ash (4.8 %) (Khoddami et al., 2011; Ali & Blunden, 2003 ▶). The seeds are additionally containing great amount of various vitamins and minerals like Cu, P, Zn and Fe and so forth (Ashraf, Ali, & Iqbal, 2006 ▶). The seeds contain carotene which is changed over by the liver to vitamin A (Kanter et al., 2005). Root and shoot of the plant are reported to contain vanillic acid (Nickavar et al., 2003 ▶; Bourgou et al., 2008 ▶).

**Figure 1:**
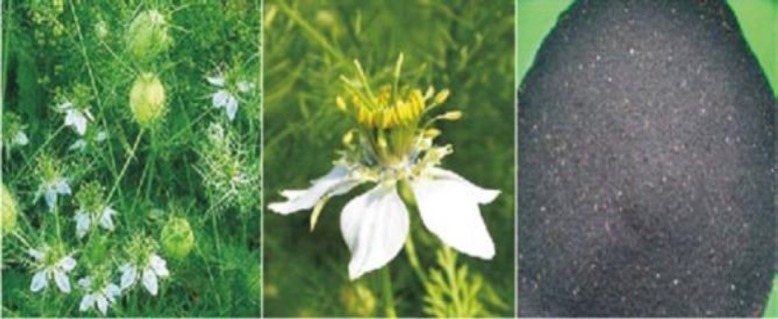
*Nigella sativa*(whole plant, flower and seeds) (Ahmad et al., 2013

The seeds are reported to contain a fatty oil rich in unsaturated fats, mainly linoleic acid (50-60%), oleic acid (20%), eicodadienoic acid (3%) and dihomolinoleic acid (10%) (Ashraf et al., 2006 ▶). Saturated fats including palmitic, stearic acid are up to around 30%(Nickavar et al., 2003 ▶). Other compounds are esters of unsaturated fatty acids with C15 and higher terpenoids, esters of dehydrostearic and linoleic acid, aliphatic alcohol and β-unsaturated hydroxy ketone (Morikawa et al., 2004). α-sitosterol is a major sterol, which represents 44% and 54% of the aggregate sterols in Tunisian and Iranian breeds of black seed oils respectively, followed by stigmasterol (6.57-20.92% of total sterols) (Cheikh-Rouhou et al., 2008; Atta, 3003 ▶). 

Examples of other reported chemical components includes nigellone, avenasterol-5-ene, avenasterol7-ene, campesterol, cholesterol, citrostadienol, cycloeucalenol, gramisterol, lophenol, obtusifoliol, stigmastanol, stigmasterol-7-ene, β-amyrin, butyrospermol, cycloartenol, 24-methylene-cycloartanol, taraxerol, tirucallol, 3-O-[β-D-xylopyranosyl (1.3)-α L-rhamnopyranosyl(1.2)-β-L-arabino-pyranosyl]-28-O-[ β-L-rhamnopyranosyl (1.4)-β-D-glucopyranosyl(1.6)-β-Dgluco- pyranosyl] hederagenin, hederagenin glycoside, melanthin, melanthigenin, bitterprinciple, tannin, resin, protein, reducing sugar, glycosidalsaponin, 3-O-[β-D-xylopyranosyl (1.2)-α-L-rhamnopyranosyl-(1.2)-β-D-glucopyranosyl]-11-methoxy-16, 23 dihydroxy-28-methy-lolean-12-enoate,stigma-5, 22-dien3-β-D-gluco-pyranoside, cycloart-23-methyl-7, 20, 22triene- 3., 25-diol, nigellidine-4-O-sulfite, N. mines A3,A4, A5, C, N. mines A1, A2, B1, and B2 (Morikawa et al., 2004 ▶).

***Nigella sativa*** **and the nervous system**

NS is useful to treat a variety of diseases of the nervous system. In this study, the effects of this plant on these diseases will be described.

***Nigella sativa*** **, anxiety and depression**

Depression is the second most common chronic disease throughout the world. It is estimated that about half of the patients are unaware of their disease or their disease is miss-diagnosed (Sharp & Lipsky, 2002 ▶). Anxiety is also a complicated disorder in human and animals which may lead to a wide range of problems in the central nervous system (CNS). It has also been reported that anxiety affects one-eighth of the population and in sever forms it has debilitating effects on the quality of life (Azizi-Malekabadi et al., 2015 ▶).

Depression and anxiety disorders are different; however, individuals with depression regularly encounter symptoms similar to those seen in anxiety disorder(Barbee, 1998 ▶).

In animal studies, elevated plus maze is a well-known research tool in neurobiological anxiety research and is used as a screening test for putative anxiolytic or anxiogenic compounds (Pellow et al., 1985). Also, the open field test is an experiment used in scientific researches to assay general locomotor activity levels, anxiety and sometimes depression in rodents(Denenberg, 1969). Also, forced swimming test is another test focusing on rat's reaction to the danger of suffocation and its results are translated as powerlessness due to negative mood. It is usually used to gauge the adequacy of antidepressants (Petit-Demouliere et al., 2005). 

Following four weeks of daily administration, NS oil (NSO) showed an increment in open field activity (Perveen et al., 2009). The animals also had a better performance when tested in elevated plus maze (Perveen et al., 2009). An oral administration of NSO raised brain levels of 5-hydroxytryptamine (5-HT), but the levels of brain hydroxyindole acetic acid (5-HIAA) significantly reduced (Perveen et al., 2009). Likewise, brain and plasma levels of tryptophan increased after repeated oral administration of NSO(Perveen et al., 2009). TQ has also shown an anti-anxiety-like effect in mice through modulation of γ-aminobutyric acid (GABA) and nitric oxide (NO) levels in the brain or plasma (Gilhotra & Dhingra, 2011). 

In another study, mice were subjected to 6 h immobilization in order to experience stressed conditions and the role of GABAergic and nitriergic modulation in the anti-anxiety effect of TQ has been investigated. TQ (10 and 20 mg/kg) produced significant anti-anxiety effects in unstressed mice without altering nitrite levels, but only the higher dose (20 mg/kg) of TQ increased the GABA content in unstressed mice. In stressed mice, TQ (20 mg/kg) showed anxiolytic effects with a significant reduction in plasma nitrite and brain GABA content. Pre-treatment with methylene blue improved the anti-anxiety effect of TQ in both unstressed and stressed mice. Hence, an association between NO-cGMP and GABAergic pathways in the anxiolytic-like activity of TQ has been proposed (Gilhotra and Dhingra, 2011). The results of the our previous study also showed that injection of 200 and 400 mg/kg of hydro-alcoholic extract of NS prevented lipopolysaccharide-induced depression-like behavior in rats(Hosseini et al., 2012), which confirmed the anti-depressive effects of the plant and suggested that the effects might be due to its anti-inflammatory properties. It is also concluded that NS, NSO and TQ improve anxiety and depression. It seems that the effects are related to the effects of GABA, NO and 5-HT.

***Nigella sativa*** **, neurotoxicity and neurodegeneration**

The term "neurotoxicity" alludes to harm to the brain or peripheral nervous system caused by exposure to natural or man-made toxic substances (Grandjean & Landrigan, 2006 ▶). These poisons can change the action of the nervous system in ways that can upset or kill the neurons (Adewale et al., 2015 ▶).

In an *in vitro* study, TQ (10 mM) protected cultured hippocampal and cortical neurons of embryos of Wistar rat brain against neurotoxicity and cytotoxicity induced by Alzheimer’s disease–specific amyloid beta (Alhebshi et al, 2013 ▶). Lewy bodies are anomalous totals of proteins that develop inside the nerve cells in Parkinson's disease (PD) and Lewy body dementia (Spillantini et al., 1997 ▶). They are recognized under a microscope when histology is performed on the brain(Dickson et al., 1996 ▶). Alhebshi et al. reported the protective effects of TQ (100 nM) against the synaptic toxicity of α-synuclein, which is accumulated in the brains of patients with Parkinson's disease and dementia with Lewy bodies (Alhebshi et al., 2013 ▶). 

In an *in vitro* study, El-naggar et al. utilized three concentrations of NS extract (2.5, 25, and 250 μg/mL) and found that NS significantly improved neuronal cell viability compared to untreated cerebellar neuron cell culture and protected against beta-amyloid protein intoxication (El-Naggar eta l., 2010). TQ (0.1 and 1 µM) pre-treatment repressed amyloid-beta-induced apoptosis of cultured cerebellar granule neurons (CGNs) via both extrinsic and intrinsic caspase pathways(Ismail et al., 2013). Hence, the ﬁndings of these studies recommend that TQ can prevent neurotoxicity and amyloid-beta-induced apoptosis. 

PC12 is a cell line which is derived from a pheochromocytoma of the ratadrenal medulla, and has an embryonic starting point from the neural crest (Greene & Tischler, 1976). NS extract (15.62–250 μg/ml) and TQ (1.17–150 μM) protected PC12 cells against cytotoxic agents via attenuation of oxidative stress (Mousavi et al., 2010). Likewise,TQ (10, 15, 25 and 35 μM) had a defensive role against ethanol-induced neuronal apoptosis in primary rat cortical neurons(Ullah et al., 2012).

Oxidative stress plays an important role in the advancement of multiple sclerosis (MS) (Bielekova & Martin, 2004). In an experimental autoimmune encephalomyelitis (EAE) mice model which mimics human MS, it was shown that administration of TQ was almost 90% preventive and 50% curative due to its antioxidant effects (Mohamed et al., 2009 ▶).

Neuroprotection refers to the relative preservation of neuronal structure and/or function (Casson et al., 2012 ▶). In the case of an ongoing insult (a neurodegenerative insult), a relative preservation of neuronal integrity implies a reduction in the rate of neuronal loss over time (Casson et al., 2012 ▶).

In another study, NS enhanced the structure and the thickness of the olfactory epithelium and lessened the lipofuscin auto-fluorescence when it was administered at a dose of 40 mg/kg/day for two months (Eltony & Elgayar, 2013 ▶). It additionally weakened the diminishment in cytoplasmic basophilia and the aggregation of lipofuscin pigment and the neurofibrillary tangles in both mitral and pyramidal cells (Eltony & Elgayar, 2013 ▶). These perceptions show that utilization of NS could be of worth in enhancing the basic changes of the peripheral and central main olfactory organs, which happen along with aging.

The effects of TQ on neuronal toxicity induced by 6-hydroxydopamine (6-OHDA) has also been reported. Unilateral intrastriatal 6-OHDA-lesioned rats showed a reduction in the number of neurons of the substantia nigra pars compacta (SNC). Pre-treatment with 5 or 10 mg/kg of TQ (p.o.) for three times with an interval of 24 h significantly prevented loss of SNC neurons (Sedaghat et al., 2014 ▶). In another study, oral administration of NS at a dose of 400 mg/kg/daily for 30 days started just after trauma to the sciatic nerve of the rats. NS markedly reduced degeneration of neurons after trauma and the count of neurons in the NS-treated was higher than that of untreated rats (Javanbakht et al., 2013). It was also reported that 400 mg/kg of NS and 50 mg/kg of TQ when administered once a day orally for 12 weeks, protected against chronic toluene-induced neurodegeneration in the rat hippocampus (Kanter, 2008).

Stroke still remains a challenge for the researchers and scientists todevelopideal drug. Neuroprotective effects of aqueous and hydro-alcoholic extracts of NS (400 mg/ kg, orally) were evaluated in middle cerebral artery-occluded (MCAO) rats. Locomotor activity and grip strength of the animals were improved and the infarct volume was also reduced in both aqueous and hydroalcoholic extracts pre-treated rats. Pre-treatment with NS extracts also prevented elevation of thiobarbituric acid reactive substance (TBARS) and reduction in glutathione and antioxidant enzymes, viz. superoxide dismutase (SOD) and catalase (CAT) following MCAO (Akhtar et al., 2012 ▶ ). In another study, chloroform and petroleum ether extract of NS seeds administered at a dose of 400 mg/kg, orally for seven days to middle cerebral artery-occluded (MCAO) rats for its antioxidant role in cerebral ischemia. The chloroform and petroleum ether extract of NS showed antioxidant, free radical scavenging, and anti-inflammatory properties (Akhtar et al., 2013 ▶).

It has also been reported that administration of TQ (5 mg/kg/day, orally) 5 days before ischemia and continuing it during the reperfusion time, prevented brain damage in a model of transient forebrain ischemia in the rat hippocampus (Al-Majed, Al-Omar, & Nagi, 2006 ▶). The study also showed that TQ stimulated resistance to oxidative stress by decreasing the elevated levels of MDA, glutathione (GSH) contents, CAT and SOD (Al-Majed et al., 2006 ▶). 

A protective effect for TQ was also reported in 1-methyl-4- phenylpyridinium (MPP)-treated primary dopaminergic cultures and a primary Parkinson’s disease model involving rotenone and neuroinflammatory mechanisms. In this study, rotenone, a well-known insecticide, following both short (20 nmole on day 10 i.v. for 48 h) and long-term (1 nmole on day 6 i.v. for 6 consecutive days) treatment

 reduced the number of tyrosine hydroxylase immunoreactive neurons by 33% and 24% which was prevented by TQ(Radad et al., 2009).

In an animal model of subarachnoid hemorrhage, the rats were injected with 0.3 ml blood into their cisterna magna. Results showed that NSO (0.2 ml/kg, i.p.) markedly improved the neurological scores, prevented blood brain barrier permeability, and increased level of brain water content which was accompanied by improvement of all oxidant responses including MDA and glutathione, myeloperoxidase (MPO), and Na+-K+-ATPase activities (Ersahin et al., 2011). In a global cerebral ischemia model, 50mg/kg of NS extract could prevent intracellular edema and decreased edematous astrocytes in the hippocampus tissue of the brain (Hobbenaghi et al., 2014 ▶).

As studies have shown, NS hydro-alcoholic extract, NSO and TQ have protective effects against neuronal damage and neurotoxicity. 

***Nigella sativa*** **, drug tolerance and withdrawal**

Physiological tolerance or drug tolerance is regularly experienced in pharmacology, when a subject's response to a particular medication and its concentration is diminished, requiring an increase in concentration to achieve the desired effect(Malenka et al., 2009 ▶). It has been well documented that repeated administration of opiates leads to development of tolerance and dependence (Hosseini et al., 2007 ▶; Hosseini et al., 2009 ▶; Karami & Zarrindast, 2011 ▶).

Tramadol is an opioid which is used to treat moderate to moderately severe pain (Adams et al., 2006 ▶). In 2011, Abdel-Zaher et al. reported that repeated administration of NSO (4 mL/kg, orally) along with tramadol (50 mg/kg, s.c. (subcutaneous)) inhibited the development of tramadol tolerance and dependence as measured by hot plate test and naloxone (5 mg/kg, i.p.)-precipitated withdrawal manifestations, respectively (Abdel-Zaher et al., 2011 ▶). They also found that NSO prevented NO over-production, increase in MDA level and reduction of glutathione and glutathione peroxidase in the brain due to repeated administration of tramadol (Abdel-Zaher et al., 2011 ▶).

**Table 1:** Different effects of NS on neurotoxicty and neurodegeneration in experimental studies

**Drug**	**Dose/ duration of treatment**	**Model**	**Mechanism/results**	**Author**
**TQ**	1.642 mg of in a 1 ml of solution made of DMSO	Embryos Wistar rat brains	Antioxidative effects against amyloid beta	Alhebshi, et al., 2013
**TQ**	100 nM	Synaptic toxicity of α-synuclein in rats	Inhibition of synaptic toxicity	Alhebshi, et al., 2013
**NS extract **	2.5, 25, and 250 μg/mL and two time points (15 and 60 min) / two time focuces (15 and 60 min)	beta-amyloid toxcification	Protection against beta-amyloid protein intoxication	El-Naggar, et al., 2010
**TQ **	0.1 and 1 µM pretreatment	Amyloid-beta -induced apoptosis of CGNs	Prevention of neurotoxicity and amyloid-beta -induced apoptosis	Ismail, et al., 2013
**NS extract and TQ**	NS extract (15.62–250 μg/ml) and TQ (1.17–150 μM) p	PC12 cytotoxicity	protect PC12 cells against cytotoxic agents	Mousavi, et al., 2010
**TQ**	in vitro study 10, 15, 25 and 35 μ M	Ethanol-induced neuronal apoptosis, in vitro study, in rat cortical neuron	protective role against ethanol-induced neuronal apoptosis in primary rat cortical neurons	Ullah, et al., 2012
**TQ **	i.p. administration from day 1 till day 50	CR-EAE Mice	Antiaoxidative effects	Mohamed, et al., 2009
**NS, given in capsules**	40 mg/kg/day for two months	Aged MOB and PC in female albino rat	Neuroprotective	Eltony, et al., 2014
**daily pretreated p.o. with TQ**	5 and/or 10 mg/Kg three times at an interval of 24 h.	6-hydroxydopamine (6-OHDA)-lesioned rats		Sedaghat, et al., 2014
**NS **	400mg/kg body weight) once a day orally/ for 30 days started just after trauma	In the trauma sciatic nerve of rats induced by placing an aneurysm clip on the left leg		Jaavanbakht, et al., 2013
**NS and TQ**	NS 400 mg/kg body weight) TQ (50 mg/kg body weight) once a day orally by using intra gastric intubation/ for 12 weeks	Toluene-induced neurodegeneration in the rat hippocampus	Reduction of neurodegeneration	Kanter 2008
**Aqueous and hydro-alcoholic extracts of NS**	400 mg/ kg, orally/ for seven days	Middle cerebral artery occluded (MCAO) rats	Antioxidative effects by reduced the oxidative stress parameters	Akhtar, et al., 2012
**chloroform and petroleum ether extract of ** **NS** ** seeds**	400 mg/kg, per orally for seven days	MCAO rats	Antiaoxidative effects	Akhtar, et al., 2013
**TQ**	(5 mg/kg/day p.o.) 5 days before ischemia and continued during the reperfusion time	Transient forebrain ischemia in the rat hippocampus	Antioxidative effects by reduced the oxidative stress parameters	Al-Majed, et al., 2006
**TQ**	adminstration of 0.01, 0.1, 1, 10 µm on day 8 i.v./ for 4 days	Parkinson’s disease model involving rotenone and neuroinflammatory mechanisms	Anti-dopaminergic effects	Radad, etal., 2009
**NSO **	0.2 ml/kg, intraperitoneally	SAH rats	Antioxidative effects by reduced the oxidative stress parameters	Ersahin, et al., 2011
**NS extraction**	10 and 50mg/kg were used during surgery through IP/ during surgery	Neuronal damage induced by Global cerebral ischemia reperfusion		Hobbenaghi, et al., 2014

Another study recommended that, most likely with the supplementation of NS to methadone, it will in a roundabout way be a beginning stage to answer the question of opioid dependency and withdrawal for better retention of patients in methadone maintenance therapy (MMT) (Adnan et al., 2015 ▶).Concurrent i.p. administration of the NO synthase inhibitor, L-N (G)-nitroarginine methyl ester (L-NAME) (10mg/kg) also potentiated these inhibitory effects of NSO on tolerance which confirms that NSO probably have a role in tramadol tolerance and dependence (Abdel-Zaher et al., 2011 ▶).Likewise, it has been demonstrated thatNS500 mg reduced the opiate withdrawal syndrome from pre-treatment day-3 in patients with opioid dependence (Sangi et al., 2008 ▶).

Interaction of NS or its components with the neurotransmitters like dopamine, glutamate, acetylcholine, GABA, histamine, and NO on the rewarding properties of morphine has been reported (Jukic et al, 2007 ▶). Injection of NS extract (200 and 400 mg/kg, i.p.) 60 min before morphine administration on the conditioning days and 60 min before the post-conditioning phase, reduced the expression of morphine-induced conditioned place preference(CPP)(Anvari et al., 2012 ▶).

In general, it seems that NSO, NS and its extract can be useful in the treatment of drug tolerance. Few studies have been done about its mechanism, but it was mentioned that the effects are in part due to the antioxidant properties. It is also noted that this plant exerts its effects via interaction with neurotransmitters.

**Table 2:** A summary of all the experiments done on NS and drug tolerance and withdrawal

**Drug **	**Dose/ duration of treatment**	**Model**	**Mechanism/results**	**Author**
**NSO**	4 mL/kg, p.o.	Tramadol-dependent mice	Blockade of NO overproduction	Abdel-Zaher, et al., 2011
**NS**	500 mg	Patients with opioid dependence		Sangi, et al., 2008
***Hydro-alcoholic *** **extract of NS**	200 and 400 mg/kg, i.p./ 60 min before morphine administration on the conditioning days and 60 min before the post-conditioning phase	Morphine-induced CPP, rats	Interaction of NS and glutamatergic system	Anvari, et al., 2012

***Nigella sativa*** **and learning and memory impairments**

As an established historical and religion-based remedy for a wide range of health problems, NS is one of the herbal medicines that is being actively investigated and is thus gaining worldwide recognition (Goreja, 2003). Individuals in different parts of the world (e.g. Bangladesh) usually take NS alone or the oil of NS with either honey or boiled mint for various health benefits such as memory improvement (Sharrif, 2011).

 A relationship between memory impairment and increased oxidative stress in the brain has been well documented (El Sherbiny eta l., 2003; Eun et al., 2008). Since oxidative stress is characterized by an imbalance in production of reactive oxygen species (ROS) and antioxidative defense, both are considered to have a noteworthy part during the time spent age-related neurodegeneration and cognitive decline (Gella & Durany, 2009)and in this manner, plants like NS which have antioxidant properties may counteract further neurodegeneration and memoryimpairment. It has also been proposed that the improving effects on memory, cognition and attentiveness in NS-treated elderly individuals are due to anti-cholinesterase property of NS (Yassin, 2005).

A previous study demonstrated that chronic oral administration of NSO could enhance the consolidation and recall capability of stored information and spatial memory in diabetic animals (Jalali and Roghani, 2009). Administration of extract of NS (200 or 400 mg/kg, i.p.) for two weeks could avert scopolamine-induced memory deficit in rats, as the animals showed better execution in passive avoidance tests and diminished acetylcholinestrase (AChE) activity in the hippocampus and cortex tissue of the brain (Hosseini et al., 2015). 

A recent study by El-Marasy and his colleagues (El-Marasy et al., 2012)revealed that oral pre-treatment with NSO 1 ml/kg significantly reversed the amnesic effect of scopolamine-induced spatial and non-spatial working memory impairments in the T-maze alternation task and object recognition test, respectively. Memory enhancing effect of NSO might be due to its antioxidant and anti-inflammatory activities.

The Morris water maze test is frequently used to evaluate spatial learning in rodents. The test relies on distal cues to navigate from start locations around the perimeter of an open swimming arena to locate a submerged escape platform (Vorhees & Williams, 2006).The passive avoidance task is another experiment that is a fear-aggravated test used to evaluate learning and memory in rodents. In this test, subjects learn to avoid an environment in which an aversive stimulus (such as a foot-shock) has been previously delivered (Ishiyama et al., 2007 ▶). Memantine is used to improve the cognitive impairments of the patients suffering from Alzheimer's disease (AD) by multiple neuroprotective mechanisms. Memantine treatment improved the cognitive performance which was presented as decreasing the escape latency and path length in the Morris water maze test and by prolonging the latency and decreasing the frequencies of entering the dark compartment in passive avoidance test (Liu et al., 2014 ▶; Ming et al., 2014 ▶). 

Using Morris water maze and passive avoidance tests, it was previously shown that treatment with hydro-alcoholic NS extract (100, 200 and 400 mg/kg) improved deleterious effects of hypothyroidism on learning and memory during neonatal and juvenile growth (Beheshti et al., 2014 ▶). Also, administration of NS 100, 200 and 400 mg/kg in drinking water during neonatal and juvenile growth, improved learning and memory of rats (Beheshti et al., 2015 ▶).

In another study, the hydro-alcoholic extract of NS (200 and 400 mg/Kg, i.p, before PTZ injection for 5 consecutive days) can improve learning and memory impairments as well as brain tissue oxidative damage after PTZ-induced repeated seizure in rats (Vafaee et al., 2015).

It has also been reported that of NS capsule (500 mg) given twice daily for nine weeks, may have positive modulatory effects on memory in elderly volunteers (Bin Sayeed et al., 2013). It was previously shown that the hydro-alcoholic extract of NS (200 or 400 mg/kg) prevented scopolamine-induced spatial memory deficits in rats; this was accompanied by inhibition of AChE activity as well as protection against brain tissue oxidative damage (Hosseini et al., 2014). Acetylcholine has an important role in the encoding of new memories (Hasselmo, 2006; Nabeshima, 1993). Enhancement of cognition and improvement of memory in groups treated with NS might be due to activation of the cholinergic system in hippocampus that plays an important role in learning and memory. NSO 60 μL/kg was force-fed daily and enhanced learning and memory abilities of the rats which presented in a significant decrease in the overall mean number of working memory error. The effects were attributed to the antioxidant and neuroprotective properties (Sahak et al., 2013). Long-term administration of NS has been shown to increase serotonin levels in the brain and improve learning and memory in rats (Perveen et al., 2008 ▶).

Many studies have been done to evaluate the effects of NS on learning and memory. In short, NSO and hydro-alcoholic extracts of NS can improve learning and memory. The proposed mechanism(s) for this effect are anti-inflammatory, antioxidant as well as anti-cholinesterase properties.

***Nigella sativa*** **and epilepsy**

The anticonvulsant effects of the aqueous extract of the seeds of NS were evaluated in experimental and clinical studies. It was demonstrated that NS extract impairs motor coordination, decreases locomotor activity but increases sleeping time (Guha et al., 2005 ▶). 

**Table 3:** A summery of all the experiments regarding the effects of NS on learning and memory impairment

**Drug**	**Dose/ duration of treatment**	**Model**	**Mechanism**	**Author**
**NSO **	Oral administration	Memory impairment in diabetic animals		Jalali, et al., 2009
**Extract of NS**	200 or 400 mg/kg of NS (intraperitoneally)/ for two weeks	Scopolamine-induced deficit memory	Redusction inacetylcholinestrase activity	Hosseini et al., 2015
**NSO **	1 ml/kg, p.o.	Scopolamine-induced deficit of spatial and nonspatial working memory impairment in rats	Antioxidant and anti-inflammatory effects	El-Marasy, et al., 2012
**hydo-alcoholic NS extract **	100,200 and 400 mg/kg in drinking water/ 8 weeks	PTU- induced learning and memory impairment	Antioxidant	Beheshti, et al., 2014s
**hydo-alcoholic NS extract**	100,200 and 400 mg/kg in drinking water/ 8 weeks	Rats		Beheshti, et al., 2014
**hydro-alcoholic extract NS**	200 and 400 mg/kg, i.p/ before PTZ injection for 5 consecutive days	Rats	Antioxidant	Vafaee, et al. 2015
**NS **	500 mg NS capsule twice daily for nine weeks	Elderly volunteers		Bin Sayeed, et al., 2013
**hydro-alcoholic extract of NS as **	200 or 400 mg/kg	Scopolamine-induced spatial memory deficits in rats	Inhibition of AChE activity	Hosseini, et al., 2014
**NSO **	Force-fed daily at the dose of 6.0 μL/100 g body weight	Twelve Sprague Dawley rats	Antioxidant and neuroprotective effects	Sahak, et al., 2013
**NS**	long-term administration	Rat	Increase in 5-HT levels in brain	Perveen, et al., 2008

In another study, pilocarpine-induced animal model of epilepsy was used and the animals were allowed for 22 days to establish the chronic phase of epilepsy. The animals were then treated with a daily oral administration of NSO (4 ml/kg) for 21 days. This study reflected the promising anti-convulsant and potent antioxidant effects of NSO in reducing oxidative stress, excitability and the induction of seizures in epileptic male Wistar albino rats (Ezz et al., 2011 ▶).

**Table 4:** Asummery of all the experiments done on NS and epilepsy

Drug	**Dose/ duration of treatment **	**Author **
aqueous seed extract of NS	Oral administration	Jalali, et al., 2009
TQ	200 or 400 mg/kg of NS (intraperitoneally)/ for two weeks 1 ml/kg, p.o.	El-Marasy, et al., 2012
TQ	100,200 and 400 mg/kg in drinking water/ 8 weeks	Beheshti, et al., 2014s
aqueous extract of NS	100,200 and 400 mg/kg in drinking water/ 8 weeks	Vafaee, et al. 2015
TQ	200 and 400 mg/kg, i.p/ before PTZ injection for 5 consecutive days	Bin Sayeed, et al., 2013
NSO	Force-fed daily at the dose of 6.0 μL/100 g body weight	Sahak, et al., 2013

***Nigella sativa*** **and pain**

The aqueous and methanol extracts of NS seeds were shown to possess potent CNS depressant effects and analgesic activities, especially depressant action in the case of the methanolic extract (Al-Naggar et al., 2003 ▶). In a neuropathic pain model of rats with chronic compressive injury of the sciatic nerve*, *NS ethanolic extract 50 mg/kg showed a significant analgesic effect (Bashir & Qureshi, 2010 ▶). Also, TQ (1.25, 2.5 and 5 mg/kg, i.p. once a day for 14 days) showed anti-nociceptive properties which was accompanied by antioxidant effects and inhibition of microglia activity (Amin et al., 2014 ▶). 

The hot-plate test evaluates the pain response in animals, similar to the tail flick test. It is used in basic pain research and in testing the effectiveness of analgesics by observing the reaction to pain caused by heat (Eddy and Leimbach, 1953). An oral administration of NSO (50-400 mg/kg) dose-dependently suppressed the nociceptive response in the hot-plate test, tail-pinch test and acetic acid-induced writhing test and in the early phase of the formalin test (Abdel-Fattah et al, 2000 ▶). The systemic administration (2.5-10 mg/kg, p.o. and 1-6 mg/kg, i.p.) and the i.c.v. injection (1-4 µg/mouse) of TQ attenuated the nociceptive response not only in the early phase but also during the late phase of the formalin test (Abdel-Fattah et al., 2000 ▶).  Results suggested that NSO and TQ produce anti-nociceptive effects through indirect activation of the supraspinal mu- and kappa-opioid receptor subtypes (Abdel-Fattah et al., 2000 ▶). In another study, NS seeds essential oil, at the doses of 100, 200 and 400 µL/kg did not exert a significant anti-inflammatory effect in the carrageenan test while i.p. injection of the same doses significantly inhibited carrageenan-induced paw edema (Hajhashemi et al., 2004 ▶). At the doses of 10 and 20 µL/ear, it could also reduce croton oil-induced edema (Hajhashemi et al., 2004). It is suggested that mechanism(s) other than opioid receptors are included in the pain analgesic effect of NS since naloxone could not reverse this effect (Hajhashemi et al., 2004 ▶).

It can be noted that the aqueous, methanol and ethanol extract of NS as well as NSO and TQ can relieve pain. The effectiveness of these drugs may be mediated through the mu- and kappa-opioid receptor. Also, this effect could be due to the antioxidant and anti-inflammatory properties and microglia inhibitory activity of this plant. 

**Table 5:** A summary of all the experiments done regarding the effects of NS on pain

**Drug**	**Dose/ duration of treatment**	**Model**	**Mechanism**	**Author**
**NS seed Ethanolic extract**	50 mg/kg	Male albino mice		Bashir, et al., 2010
**Thymoquinone**	1.25, 2.5, and 5 mg/kg, i. p./ once a day for 14 days	Neuropathic pain of rats with chronic constrictive injury of the sciatic nerve		Amin, et al., 2014
**NSO and Thymoquinone**	p.o. administration of NSO (50-400 mg/kg), The systemic administration (2.5-10 mg/kg, p.o. and 1-6 mg/kg, i.p.) and the i.c.v. injection (1-4 microgram/mouse) of TQ	Mice	Indirect activation of the supraspinal mu - and kappa-opioid receptor subtypes	Abdel-Fattah, et al., 2000
**NS seeds essential oil**	100, 200 and 400 micro L/kg i.p. injection	Carrageenan-induced paw oedema in rats	Mechanism(s) other than opioid receptors	Hajhashemi, et al., 2004

## Conclusion

This review article summarized *in vitro* and *in vivo* studies in order to report the effects of NS and its active constituents on the nervous system. According to different studies it seems that NS can affect the nervous system and related diseases. In these studies, aqueous, alcoholic and hydro-alcoholic extracts and NSO has been considered. It should be mentioned that TQ is seen to be the most useful known element of NS and can be regarded as a useful agent in the treatment of diseases of the nervous system. The results of several studies have shown that this plant can improve memory impairment, anxiety, depression, epilepsy, neurotoxicity, neurodegeneration and pain. In addition, based on the current review, it is concluded that NS, through inhibition of acetylcholinestrase enzyme and particularly due to its antioxidative effects improves nervous system diseases. It is also suggested that NS has interactions with the GABA, opioid and NO system. However, a few studies confirmed the beneficial effects of NS on epilepsy and seizures in children. The studies which were reviewed here were preliminary studies and future studies are needed to be done to evaluate the effects of the clinical use of the plant on the nervous system.
